# Experimental Study on Sand Stabilization Using Bio-Cementation with Wastepaper Fiber Integration

**DOI:** 10.3390/ma14185164

**Published:** 2021-09-08

**Authors:** Meiqi Chen, Sivakumar Gowthaman, Kazunori Nakashima, Shin Komatsu, Satoru Kawasaki

**Affiliations:** 1 Division of Sustainable Resources Engineering, Graduate School of Engineering, Hokkaido University, Sapporo 060-8628, Japan; 2 Department of Engineering Technology, Faculty of Technology, University of Jaffna, Kilinochchi 44000, Sri Lanka; gowtham1012@outlook.com; 3 Division of Sustainable Resources Engineering, Faculty of Engineering, Hokkaido University, Sapporo 060-8628, Japan; k.naka@eng.hokudai.ac.jp (K.N.); kawasaki@geo-er.eng.hokudai.ac.jp (S.K.); 4 Meiwa Seishi Genryo Co., Ltd., Osaka 532-0011, Japan; s-komatsu@meiwa-paper.co.jp

**Keywords:** microbially-induced carbonate precipitation (MICP), wastepaper fiber, mechanical properties, calcium carbonate, freeze-thaw durability

## Abstract

Recently, green materials and technologies have received considerable attention in geotechnical engineering. One of such techniques is microbially-induced carbonate precipitation (MICP). In the MICP process, CaCO_3_ is achieved bio-chemically within the soil, thus enhancing the strength and stiffness. The purpose of this study is to introduce the wastepaper fiber (WPF) onto the MICP (i) to study the mechanical properties of MICP-treated sand with varying WPF content (0–8%) and (ii) to assess the freeze–thaw (FT) durability of the treated samples. Findings revealed that the ductility of the treated samples increases with the increase in WPF addition, while the highest UCS is found with a small fiber addition. The results of CaCO_3_ content suggest that the WPF addition enhances the immobilization of the bacteria cells, thus yielding the precipitation content. However, shear wave velocity analysis indicates that a higher addition of WPF results in rapid deterioration of the samples when subjected to freeze–thaw cycles. Microscale analysis illuminates that fiber clusters replace the solid bonding at particle contacts, leading to reduced resistance to freeze–thaw damage. Overall, the study demonstrates that as a waste material, WPF could be sustainably reused in the bio-cementation.

## 1. Introduction

Due to lack of awareness of the environmental issues in the past centuries, our destructive development path has led to many irreversible changes to the earth, which are dominated by climate change, severe loss in nature, and environmental pollution [1]. Notably, in conjunction with the materials industry, the construction industry has played an essential role as one of the major natural resource consumers, contributing significantly to the environmental pressure and unsustainable development [2]. Meanwhile, the concept of technology innovation has emerged as the times require, paving a path for the future generation to construct a more sustainable society, and it weighs far more heavily than saving limited existing resources in future development [3]. Corresponding to the urgent need for sustainable technology, novel techniques and related research to soil improvement have been springing up remarkably in the construction industry [4]. Microbially-induced carbonate precipitation (MICP) has matured a great deal as a novel technique for soil improvement since it was first proposed by Stocks-Fischer et al. [5], suggesting a potential to treat the surface of porous media. Based on multidisciplinary research, MICP refers to utilizing microorganism function to induce the carbonate from urea to precipitate in the presence of calcium ions, filling the space in soils and bonding the loose particles to achieve an improvement in the soil’s performance. The biochemical reactions that happen in this process could be described as follows [6,7] (Equation (1) and (2)):

(1) $$\mathrm{CO}{\left({\mathrm{NH}}_{2}\right)}_{2}+\;2H_{2}O\;\overset{\mathrm{Urease}}{\to }\;2{\mathrm{NH}}_{4}^{+}+{\mathrm{CO}}_{3}^{2-}\;$$

(2) $${\mathrm{CO}}_{3}^{2-}+{\;\mathrm{Ca}}^{2+}\;\overset{\mathrm{Bacterial}\;\mathrm{cell}\;}{\to }\;\mathrm{Cell}-{\mathrm{CaCO}}_{3}↓.$$

Inspired by a natural phenomenon of microbial mineralization, applications of MICP have been expanding into a broader range over the past two decades. For instance, it can be an alternative to rigid structures using concrete for coastal erosion mitigation in the marine environment [8,9,10]. In terms of the construction industry, this technique is proved to have a promising future to be applied in soil improvement [11], liquefaction prevention [12], road construction [13], and slope stabilization [14]. Furthermore, self-healing concrete (bio-concrete) as a novel material now attracts more and more interest, and this bioremediation method could also be applied to cracked concrete [15]. It contributes to environmental restoration by erosion control, fugitive dust control, surface carbon dioxide sequestration, etc. [16]. Recent review work also figured out that versatile as this technique is, more joint efforts are required to evaluate the long-term performance under different environments and reduce the cost needed in the material and the process [17,18]. Existing research related to lowering the cost in MICP treatment focuses on mainly low-cost calcium resources [19], carbonate resources [20,21], and culture medium [22,23]. In terms of the durability, limited research work has been done on bio-cemented samples. Generally, the physical weathering of rock is dominated by frost wedging in cold regions with seasonal or even daily FT cycles, which cause severe rock deterioration and damages to buildings, road pavement, pipelines, etc. [24]. Considering that bio-cemented soils are often referred to as rocklike materials [25], it is highly essential to investigate the resistance against FT cycles before applying in regions such as Hokkaido (one of the coldest islands in Japan) where soils are frozen through a long winter. 

Various fiber materials have been widely used for soil improvement for more than 50 years since Vidal first proposed fiber reinforcement in 1969 [26]. Notably, the applications of some conventional synthetic fibers (such as carbon fibers, Kevlar fibers, and glass fibers) derived from by-products of the petroleum industry are proliferating rapidly. In recent years, these fibers are also introduced into MICP research and have produced many remarkable outcomes [27,28,29,30]. Although these fibers have several merits, their disadvantages include the high cost and environmental impact. In consideration of the impact on the environment and cost-effectiveness of the fibers mentioned above, some researchers investigated the utilization of natural fibers in MICP, such as plant fibers [31], animal fibers [32], and mineral fibers [33]. Plant fibers are usually preferred for their low cost. However, producing fiber resources takes a certain period to grow sufficient vegetation, and it consumes energy to pre-treat these materials physically/chemically. Therefore, this situation turns our attention to reusing waste materials, which can be a more sustainable alternative.

The objective of this study is to make an original contribution by introducing wastepaper fiber (WPF) materials into bio-cemented sand materials and evaluating the long-term durability under frozen conditions. For these purposes, the MICP-treated sand columns with varying WPF content were subjected to series of compression tests and freeze–thaw (FT) durability analysis. Meanwhile, to deepen the understanding of the mechanism, the experimental program also contains measurements of sample density, the spatial distribution of calcium carbonate content, and shear wave velocity reduction. Additionally, scanning electron microscopy (SEM) was used to obtain micro-scale observations on tested specimens.

## 2. Materials and Methods

### 2.1. Sand Characteristics

**Figure 1:**
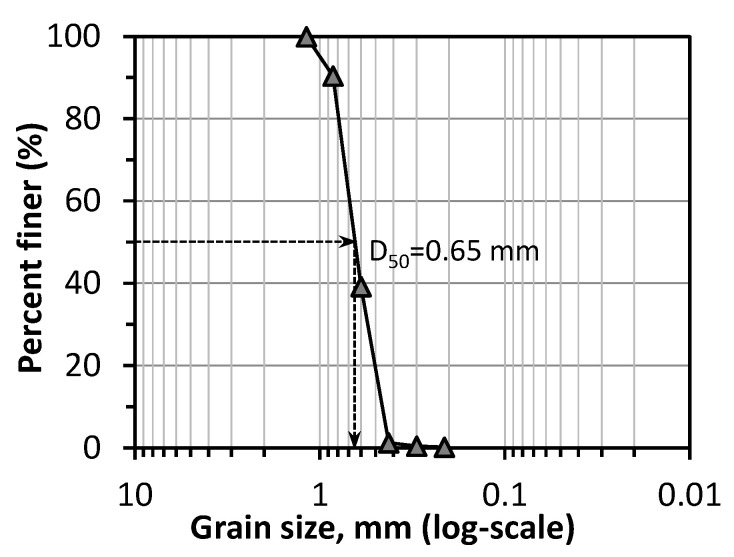
Grain size distribution of Mikawa sand.

Mikawa sand, one of the Japan standard sands, is used in this study. Figure 1 illustrates the grain size distribution of Mikawa sand. Composition analysis was conducted using a dispersive X-Ray Fluorescence (XRF) spectrometer (JSX-3100R II JOEL, Tokyo, Japan), revealing the main contents of 97.65% of SiO_2_, 0.89% of Al_2_O_3_, 0.43% of Fe_2_O_3_, 0.18% of K_2_O, 0.11% of MgO, and 0.11% of Na_2_O.

### 2.2. Wastepaper Fiber

**Figure 2:**
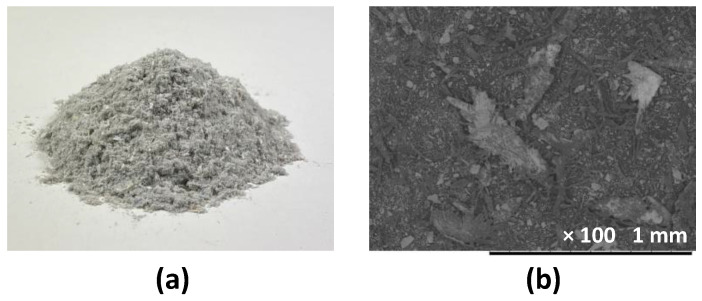
(**a**) Physical appearance; (**b**) SEM image of the WPFs.

The wastepaper fibers (WPFs) in Figure 2a were dust produced during the recycling process in a paper mill of Meiwa Seishi Genryo Company, Osaka, Japan. As these fibers are from the wastepaper, the main component was considered to be the plant’s cellulose fiber. The fibers’ length was found to be in the range of 20–500 µm by observing SEM (scanning electron microscope) images, as presented in Figure 2b. Due to the strict law on waste material management in Japan, the toxic component analysis on WPF was conducted by using the standard issued by Food and Agricultural Materials Inspection Centre (FAMIC), Tokyo, Japan. Some toxic metal elements were identified and listed in Table 1. It is worth mentioning that metals naturally exist in plants with varying contents in different species. During the process of papermaking and recycling, metals are commonly added in inks, pigments, coatings, etc. [34]. It is worth noting that these metals’ contents are lower than the standard limits, posing no threat to the environment. During the specimen preparation, the WPF was mixed into the sand without any pre-treatment.

### 2.3. Bacteria Cultivation and Cementation Media

The ureolytic bacterium used in this study is *Lysinibacillus xylanilyticus*, which is a rod-shaped Gram-positive species (8–10 µm long; 0.5–0.6 µm in diameter). It was isolated from Hokkaido, Japan. A detailed isolation process was described thoroughly in the previous work [23]. *Lysinibacillus xylanilyticus* shows a relatively high urease activity at a mild temperature, which peaks around 3.5 U/mL after 72 h of main culture (OD_600_ = 4–4.5) at 25 °C. The recent research of Gowthaman et al. [14] using *Lysinibacillus xylanilyticus* demonstrated that the column specimens were solidified successfully by MICP treatment and confirmed the feasibility of slope soil stabilization. 

As verified in the previous study, the temperature during the cultivation and specimen-curing period was set to be 25 °C. The culture medium for cultivating the bacteria and the cementation media used for the MICP treatment are listed in Table 2.

### 2.4. MICP Treatment

**Figure 3:**
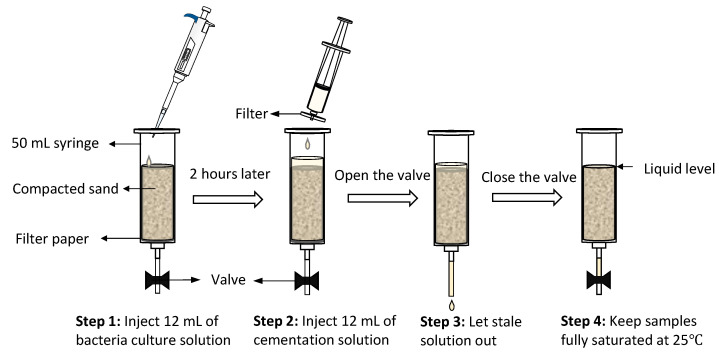
MICP treatment processes.

The barrel part of a 50 mL disposable syringe (30 mm in diameter) was used as the mold to shape and compact the sands into columns. For each specimen, 60 g of Mikawa sand was added. To prevent the sand from being washed out during treatment, the barrel’s bottom was lined with a filter paper piece before filling the mold with sand. Similarly, a filter was used to reduce the adverse effect of large organic particles in the nutrient broth on carbonate precipitation. The two-phase injection strategy adopted in this study is in accordance with previous research [14,35]. The two-week treatment could be considered as two cycles of injection, which is initiated by a 12 mL injection of bacteria culture solution (OD_600_ = 4–4.5) on the first day followed by a one-week treatment with 12 mL of cementation solution every 24 h. Each step of the treatment is explained pictorially in Figure 3.

### 2.5. Experimental Design

**Figure 4:**
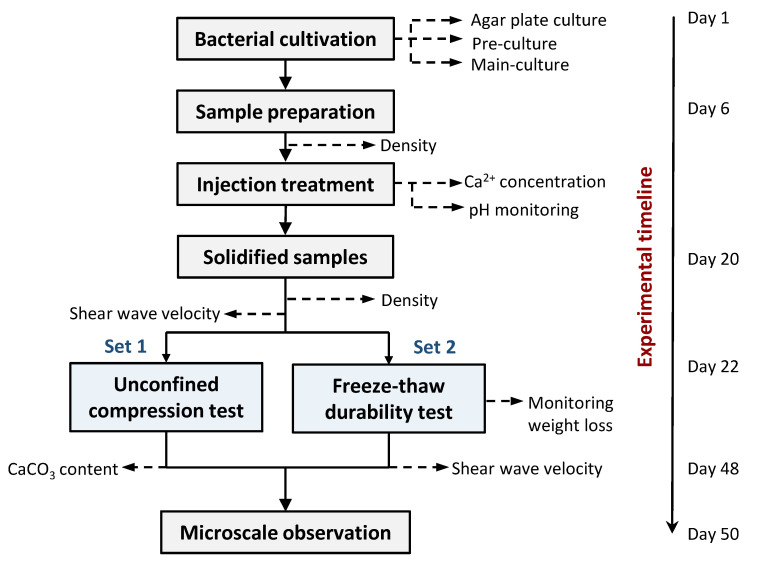
Experimental flow of this laboratory-scale study.

Based on the review work done by Gowthaman et al. [26], the fiber addition in this study was determined in the range of 0–8%, between which considerable similar research using plant fiber found an optimum addition ratio. For this reason, five cases (0%, 1%, 2%, 4%, and 8%) were prepared to investigate the feasibility of fiber reinforcement in MICP-treated sand specimens and examine the strength improvement. Meanwhile, another set of solidified specimens was subjected to the FT durability test. A schematic flow of the laboratory-scale experiments is illustrated in Figure 4.

### 2.6. Evaluation Methods

#### 2.6.1. Measurement of Shear Wave Velocity

Except for calcium carbonate content, the ultrasonic wave velocity measurement is usually conducted to characterize the MICP-treated soils without destroying the sample [36]. In this study, the SonicViewer-SX Model-5251 (OYO, Tokyo, Japan) was used to read the S-wave propagation with high accuracy. A reduction rate of shear wave velocity was calculated based on the equation given below (Equation (3)).

(3) $$\mathrm{Shear}\;\mathrm{velocity}\;\mathrm{reduction}\;\mathrm{rate}\;\left(\%\right)=\frac{V_{s}-V_{\mathrm{so}}}{V_{\mathrm{so}}}\times 100\%$$

*V*_so_ represents the initial shear velocity of the MICP-treated sample, and *V*_s_ is the velocity after 25 freeze–thaw cycles.

#### 2.6.2. Determination of Calcium Carbonate Content

The carbonate content of treated samples was measured as the total gas volume generated after a complete reaction with hydrochloride in a closed chamber system. It is a simplified method developed by Fukue et al. [37] and has been frequently used by researchers these days to estimate the carbonate content [38]. First, 2 g of dry samples and 20 mL of HCl solution (2 mol/L) are placed into a chamber separately. Then, the chamber is sealed and equipped with a pressure gauge, which indicates the chamber’s pressure change. By shaking the device, samples are allowed to mix and react with the acid solution. Then, the final stable pressure value is used as a parameter to estimate the samples’ carbonate content. After transferring the gas volume to the mass of the carbonate through a developed calibration curve, the carbonate content is obtained using Equation (4).

(4) $$\mathrm{Carbonate}\;\mathrm{content}\left(\%\right)=\frac{m_{c}}{m_{s}-m_{c}}\times 100\%$$

where *m*_c_ represents the weight of calcium carbonate in the tested sample, and *m*_s_ is the tested soil sample’s total weight.

#### 2.6.3. Unconfined Compression Test

An unconfined compression test, a standard laboratory test, was conducted to derive the treated sand samples’ unconfined compressive strength. Samples were trimmed to meet the standard specification requirement (a length-to-diameter ratio between 2.0 and 2.5), and the machine used in this test is an INSTRON 2511-308 load cell (Norwood, MA, USA), following the ASTM D7012-14e1 [39]. The stress was measured continuously at a constant strain (ramp rate: 0.036 mm/min). The axial strain and unconfined compressive strength are calculated as follows (Equation (5)),

(5) $$\varepsilon =\frac{∆l}{L_{0}},\;\sigma =\frac{P_{\max}}{A_{0}}$$

where ∆*l*: change in axial length, *L*_0_: initial length of the sample, *P*_max_: the maximum load, *A*_0_: initial cross-section area.

#### 2.6.4. Freeze–Thaw Test

The freeze–thaw test conducted in this study is in line with the procedures given in ASTM D 5312-04 [40]. Water expands around 9.05% in volume when it freezes, which exerts pressure and causes damages to the surrounding object [24]. The purpose of the FT test is to evaluate the resistance to frost wedging that occurs in rocks. Similarly, in this study, it is conducted to investigate the FT durability of "artificial rock." MICP-treated samples were subject to sufficient water rinsing to ensure no precipitation happens during the test. Prior to testing, the treated specimens were completely immersed in distilled water for 6 hours to keep samples in a state of full saturation. Each cycle was started with 12 h freezing at −20 °C and ended with 12 h thawing in a water-saturated condition at 25 °C. In total, samples went through 25 FT cycles. All the procedures followed the previous research of Gowthaman et al. [41].

#### 2.6.5. Scanning Electron Microscopy Analysis

Microscale observation is essential to analyze the mechanism of microbially induced carbonate precipitation. The instrument used in this study is Miniscope TM 3000 (Hitachi, Tokyo, Japan). Samples were observed in high vacuum and dry conditions.

## 3. Results and Discussion

### 3.1. Solidification Process Monitoring

**Figure 5:**
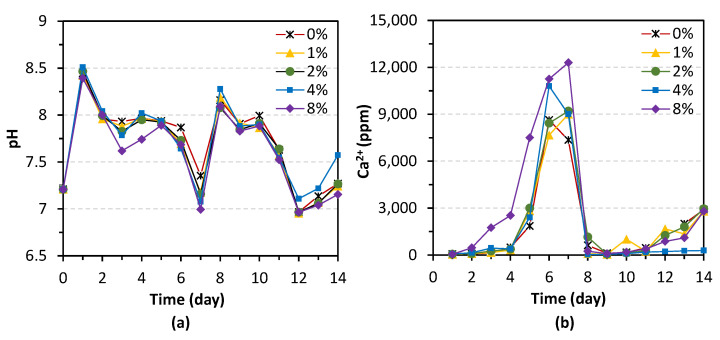
Process monitoring of (**a**) pH and (**b**) concentration of calcium ions during MICP treatment.

During the solidification process, the changes of pH and calcium ion concentration were measured from the stale outflow every 24 h. The mean values obtained from two samples are presented in Figure 5. Theoretically, the pH in the solution increases with the hydrolysis of urea. Thus, pH and the remaining calcium ion changes could be an indicator of urease activity. Since bacteria culture was only injected once a week, how long the bacteria could continue to induce precipitation is of great importance to evaluate the efficiency of the MICP. The results in Figure 5 shows that after 5 days from the bacterial injection, the urease activity dropped but remained at a certain level, which suggests that the two-week treatment strategy adopted in this study is of high effectiveness.

### 3.2. Properties of Prepared Samples

After two-week MICP treatment, solidified samples were taken out from the syringe. Using a hot knife (Goot HOT-30R, Hiroshima, Japan) to cut the syringe minimized the disturbance to samples. All samples were appropriately trimmed to shape the sample into a standard size (30 mm in diameter, 60 ± 2 mm in length) using a rock-cutting machine. Before the compression test, a grease coating was done to make the surface of the samples meet the required flatness. 

#### 3.2.1. Sample Density

**Figure 6:**
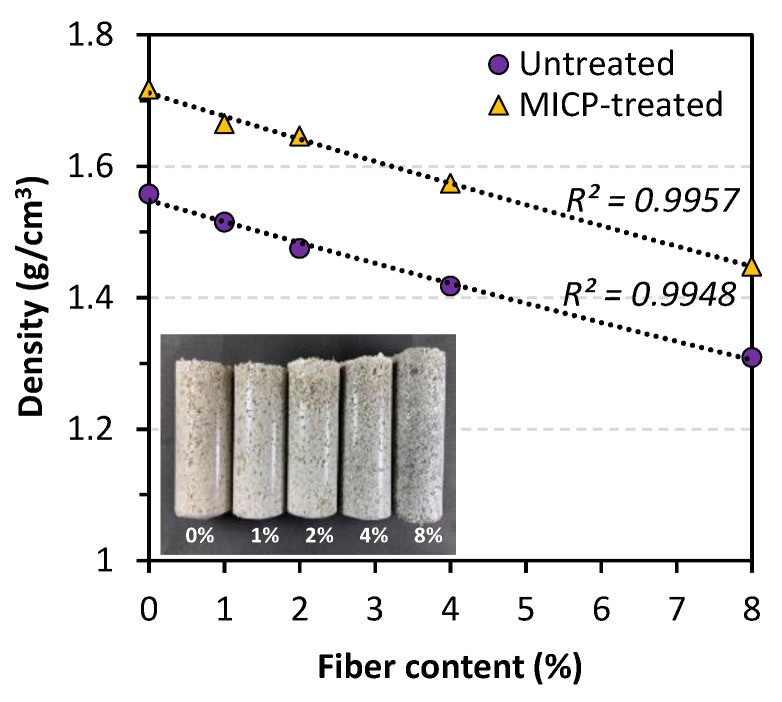
Comparison of sample density before and after MICP treatment.

The picture embedded in Figure 6 shows that the volume of high fiber content samples increased significantly, indicating a decreasing density. To confirm the effect of fiber addition on sample density, a quantitative measurement was conducted before and after the MICP treatment (shown in Figure 6). Before treatment, compared to the control case, sample density dropped around 16% when 8% of fiber added. It is encouraging to find that after MICP treatment, all samples gained an increase of around 10% in density.

#### 3.2.2. Spatial Distribution of Calcium Carbonate Content

**Figure 7:**
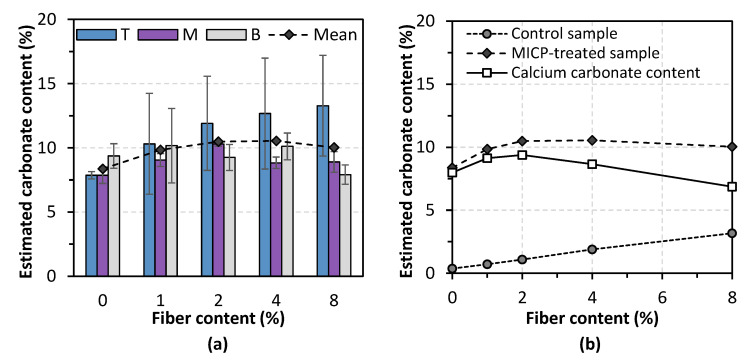
Estimated calcium carbonate content of MICP-treated samples: (**a**) carbonate content from different depths; (**b**) estimated carbonate content.

In terms of calcium carbonate, the total amount of precipitation and its spatial distribution are two critical evaluations governing the effectiveness of MICP. Figure 7a presents the calcium carbonate content measured at three points (top, middle, and bottom) along the depth. To improve the accuracy of carbonate estimation, the effect of localized carbonate in fibers was reduced from the total measurement. The solid line in Figure 7b presents an accurate carbonate content. The control case obtained a total precipitation of 8%. It is encouraging to find that this content increased by around 15% when adding a small number of fibers (1–2%), while higher addition led to a decreasing carbonate content. Closer observation of the carbonate content along the column depth gives an interpretation of the results mentioned above. In the control case, bacteria cells tend to settle down to the bottom part or flush out due to gravity and the large pore space between coarse sand particles. This situation was changed when fibers were involved. As the bacteria solution filtered through the sample from top to bottom, bacteria cells were trapped by randomly distributed fibers along the way downward. In the research of Zhao et al. [29], it was reported that 2% of activated carbon fiber felt powder contributed to more than 50% of the bacterial retention rate, which was 2.8-fold that of the control samples. In this study, the optimum fiber addition favoring the calcium carbonate precipitation yield was found to be around 2%.

### 3.3. Unconfined Compressive Strength

**Figure 8:**
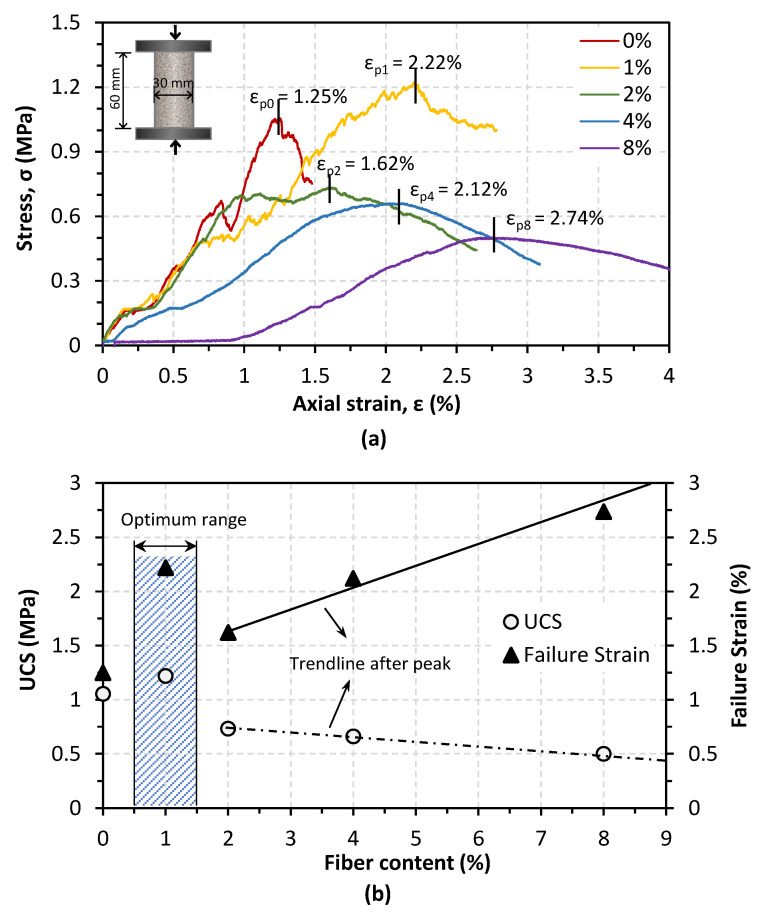
(**a**) Stress–strain behaviors of MICP-treated samples; (**b**) relationship between fiber content with UCS and failure strain.

Figure 8a demonstrates the unconfined compressive strength (UCS) result derived from five treated samples. It can be seen that the control sample (0%) achieved a strength of around 1 MPa, and it exhibited a strain-softening behavior with a sharp decrease of post-peak strength. UCS increased to 1.2 MPa when adding 1% of fibers; meanwhile, a lower peak loss was observed, indicating a strain-hardening trend. The strength gain turned out to be less when the fiber addition became more than 1%, but the samples’ ductility was enhanced significantly. Since bio-cemented sand is typically a brittle material, fiber addition provides an essential improvement for bio-cemented sand samples on strength and stress–strain behaviors [30]. It is worth noting that there is a delicate balance between strengthening and softening in terms of peak strength. From the trendlines in Figure 8b, it can be seen that the failure strain increased with increasing fiber content, implying a transformation from a brittle material (strain-softening behavior) into a more ductile one (strain-hardening behavior). Overall, it illustrates that the increase in fiber addition resulted in a decrease in compression strength.

Previous research by Li et al. [28] found an optimum fiber (polypropylene fiber, with a diameter of 0.1 mm and a length of 12 mm) content of 0.2–0.3% for significantly improving bio-cemented Ottawa silica sand. It was attributed to the nonuniformly distributed bacteria resulting from fibers’ hindrance, which led to more precipitation on the fiber surface than the contact between soil particles. In this study, the results of precipitated calcium carbonate precipitation are highly consistent with this explanation. Another research study by Fang et al. [42] using polyester fibers with similar sizes also found an optimum fiber content of 0.2%. The research of Zhao et al. [29] investigated the effect of six different additions (from 0% to 2%) of activated carbon fiber felt, which was smashed waste with a uniform grain size from 140 to 173 µm, on enhancing the strength of bio-cemented sand, and results showed that there was no optimum fiber content in this range. In another research study of Zhao et al. [43], they compared four types of different fibers (with the same length of 4 mm) regarding mechanical behaviors and found that the optimum fiber content was 1 ± 0.5%. Therefore, this discrepancy in the optimum fiber content was mostly dependent on the fiber length. In this study, fibers were waste materials with a chief component of cellulose fiber. Thus, it is impossible to control the fiber size in a certain range to make it a uniform material. In terms of strength improvement, a small fiber addition has the potential to reinforce the MICP-treated sand.

### 3.4. Freeze–Thaw Durability

#### 3.4.1. Visible Sign of Loss

**Figure 9:**
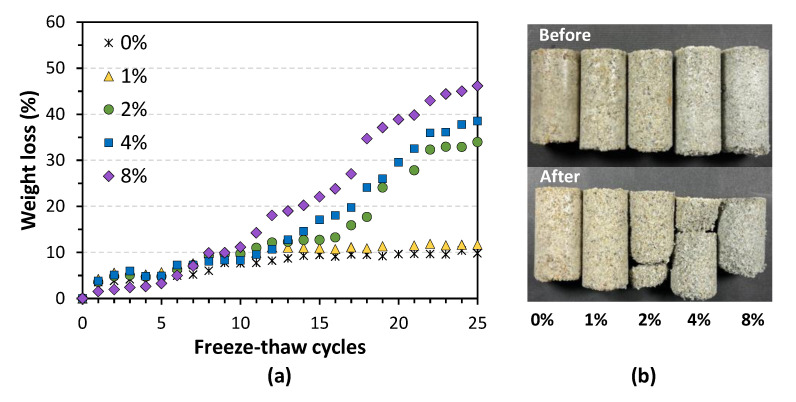
Sign of loss: (**a**) monitoring of weight loss of MICP-treated samples during 25 FT cycles; (**b**) physical appearance before and after 25 FT cycles.

As shown in Figure 9a, samples’ mass loss was monitored every 24 h. Figure 9b compares samples in terms of appearance before and after the 25 FT cycles. During the test, visible signs of loss manifested themselves after 5 FT cycles. What is striking here is that minor damage was visible on low fiber addition (0%, 1%) samples, whereas extensive damage was observed in samples with high fiber addition (2%, 4%, and 8%). It is worth noting that cracks developed in the 2%, 4%, and 8% cases and finally broke the samples into pieces. Small pieces were considered to be the loss, and big pieces remained. The weight loss data shown in Figure 9a confirmed that with the fiber addition increasing, treated samples became more prone to erosion caused by daily FT cycles. It can be found that the weight loss of the 1% case is 12%, which is similar to that of the control sample (less than 10%), which further corroborated the UCS results mentioned above. For these two samples, particle detachment was almost finished by the end of 10 cycles, thus showing a negligible increase in weight loss after the remaining 15 cycles. Some soil improvement research reported that after eight FT cycles, most of the soils do not change much, both physically and mechanically [44]. On the other hand, samples with high fiber addition appear to increase linearly during the test. At the end of this test, the weight loss of these samples was 46%, 39%, and 34%, respectively. Compared to the research of Gowthaman et al. [41], in which three different levels of bio-cemented samples were examined, the weight loss of the 8% addition case was similar to that observed in the cemented samples with 12–14% carbonate precipitation. As mentioned in the UCS results, a high fiber addition led to increased calcium carbonate content while decreasing the strength. Therefore, in this study, the effective bonding between sand particles was limited in the cases with high fiber addition, rendering the samples vulnerable when exposed to daily FT cycles.

#### 3.4.2. Mechanical Deterioration

**Figure 10:**
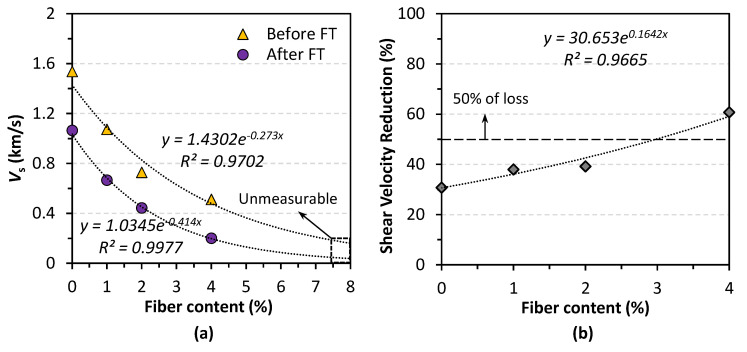
(**a**) Before–after comparison of shear wave velocity; (**b**) shear wave velocity reduction rate.

Figure 10a contrasts the shear wave (S-wave) velocity of samples before and after 25 FT cycles. As the solid line in Figure 10a shows, the shear wave velocity of treated samples tends to decrease with the fiber addition increasing, and when the addition reached 8%, it is no longer measurable, indicating a significant reduction in density. This drop was 30% on the 1% case in comparison with the control sample. Moreover, with 2% and 4% fiber addition, the shear wave velocity dropped by more than 52% and 66%, respectively. The shear wave velocity after the FT test was depicted in the dashed line shown in Figure 10a, revealing an identical tendency as that before the test. As the waves traveling through the contacts between particles, the total number of contacts significantly affects the velocity [36]. Since many contacts have been broken during the FT test, it exhibited a reduction in the shear wave velocity. This result suggests that all samples have been damaged by inner microcracks to different levels during the FT test.

The rate of shear wave velocity reduction is illustrated in Figure 10b. What can be seen in this figure is that the shear wave velocity reduction, which was calculated by Equation (3), increased almost linearly with the increasing fiber addition. Notably, a decrease of 60% was found in the 4% case, which doubled the control sample’s reduction. Similar cases in the research of Gowthaman et al. [41] have observed a shear velocity reduction of up to 20%, and these samples became unmeasurable after 15 FT cycles. 

In conjunction with the UCS results mentioned before, these results further proved that fibers have an adverse on effective bonding and did not help resist freeze–thaw erosion. Some studies related to MICP-treated samples’ durability have reported a good resistance against freeze–thaw with a loss in strength of 10% after 10 FT cycles [45,46]. On the other hand, similar research of fiber-reinforced bio-cementation by Liu et al. [47] has reported that fiber-reinforced MICP samples maintained a higher residual strength, whereas the reinforced sample’s strength loss rate after 15 FT cycles was found to be around 60%. This conclusion is consistent with the results of this study to some extent.

### 3.5. Microscale Observation

**Figure 11:**
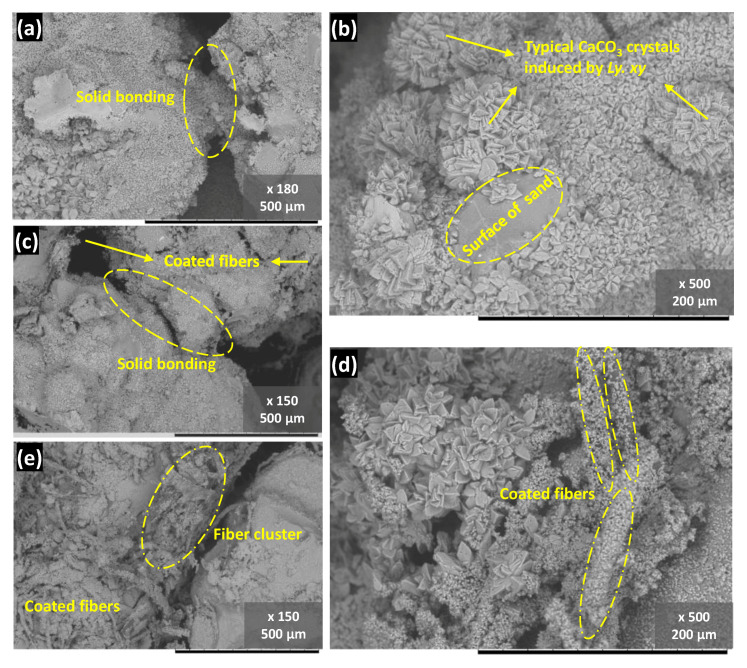
SEM images of representative MICP-treated samples with (**a**,**b**) 0%; (**c**) 1%; (**d**) 2%; and (**e**) 4% of fiber content.

To clarify the mechanism of reinforcement and softening effect of fibers with different addition, SEM analysis was conducted on MICP samples after a two-week treatment, which is shown and compared in Figure 11. It can be seen in Figure 11a that the solid bonding between particles is apparent, with each particle completely covered by the carbonate precipitation. This type of reliable bonding appears to be unaffected by the 1% fiber addition, since no significant difference was detected. As the WPF addition increased (shown in Figure 11c–e), more and more precipitations were observed around the fibers, coating and binding them together. Notably, a high fiber addition had an adverse influence on the effective bonding, which was presented as strong carbonate bonding replaced by a weakened bonding of clustered fibers between sand particles in Figure 11e. Overall, a high fiber addition led to a decreased improvement ratio, whereas a small quantity of fiber provides tensile resistance and enhances the strength to some extent.

**Figure 12:**
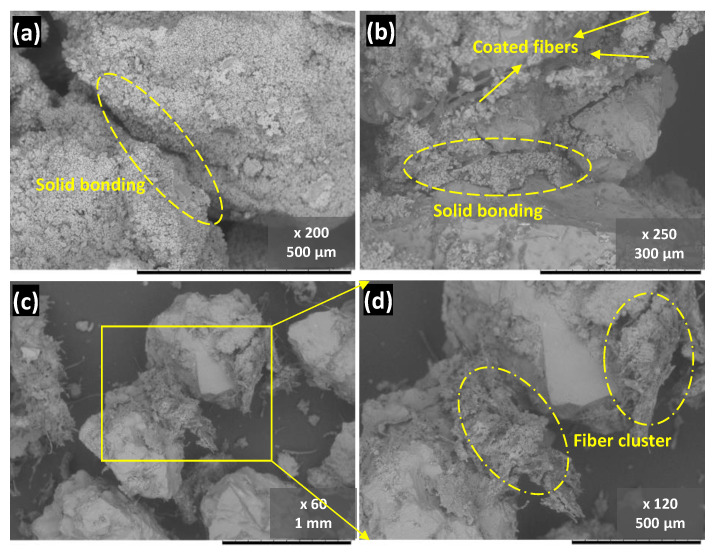
SEM images of representative treated samples after 25 FT cycles: (**a**) 0%; (**b**) 1%; and (**c**,**d**) 8% fiber content.

SEM observation after 25 FT cycles is shown in Figure 12. Contrary to expectations, these images did not distinguish themselves much from those of the treated samples. For the 1% case, as small a quantity as it is, the bonding between particles remained, revealing that a small amount of fiber did not significantly affect the durability. On the other hand, in the sample with 8% fiber addition, as shown in Figure 12c,d, the bonding bridge was loosened. This evidence suggests a probable interpretation of the destruction in samples with high fiber content: the contact part bonding was displaced by the fiber clusters that held more water during the FT cycles, hence exerting higher pressure on the surrounding particles as the water expands. It indicates that FT cycles further escalated the damage on these weak bonding areas dominated by clustered fibers. Cheng et al. [45] have concluded that the soil porosity, pore size, and bonding behavior in the MICP-treated soil matrix are three main contributing factors that govern FT durability. In this study, the bonding behavior was significantly altered by introducing fiber, which explains why samples with fibers are more prone to FT erosion. Combining the findings mentioned above suggested that a small quantity of WPF addition contributed to improving conventional MIPC-treated sand’s strength and failure pattern. Although the strength after 25 FT cycles did not reach the desirable improvement, this study figured out that a small fiber addition is enough for the fiber-reinforced bio-cemented sand, and it has the potential to be improved further.

## 4. Conclusions and Recommendations

1. This study was primarily designed to investigate the feasibility of introducing WPF onto bio-cementation and to examine the mechanical properties and durability characteristics of treated sand samples. Based on outcomes from the experimental study, the following conclusions are drawn. WPF was responsible for increasing the yield of calcium carbonate by retaining more bacteria cells during surface percolation. As WPF content increased, more precipitation was observed at the top part of the specimen.
2. A small quantity of WPF addition enhanced both the peak and the residual strengths of the MICP-treated specimens. Basically, increasing the fiber content contributed to lower strength gain and higher failure strain, indicating a great improvement in the samples’ ductility.
3. FT test results showed that experiencing 25 freeze–thaw cycles rendered samples with high fiber content vulnerable. However, the small WPF addition case was quite comparable with the control specimens. The shear wave velocity revealed that all the specimens underwent a reduction ranging from 30% to 60%, implying a reduced density caused by losing reliable contacts inside.

In general, these findings have verified that the WPF could be a promising and sustainable material for improving conventional bio-cementation and reducing the cost of treatment. However, to develop a more comprehensive understanding of fiber-reinforced bio-cementation, more experimental investigations are required. 

As an experimental work focusing on feasibility investigation, some assessments adopted in this study might not be appropriate for field application. For example, current data obtained from unconfined compression tests quickly developed the relationship be-tween fiber content and strength improvement. However, to understand the stress–strain relationship in a more realistic manner, assessments such as triaxial tests are needed. Therefore, it is recommended to conduct tests under more realistic conditions. Since a decreasing FT resistance was observed in samples with fiber addition, it is necessary to figure out how to improve the resistance. Furthermore, the FT durability test is not enough to provide a compressive understanding of their durability characteristic. Therefore, more tests under different erosion conditions are recommended for future work.

## Figures and Tables

**Table 1:** Basic properties and proportions of toxic metal elements in WPF.

Basic Properties		Composition of the Metal Element Based on Dry Weight (mg/kg)									
Moisture Content%	pH ^1^	Cd	Pb	As	Hg	Cr	Ni	Se	Cu	Zn	Al
7.70%	8.6	0.16	0.57	0.51	0.01	2.61	1.3	<0.05	23.73	8.99	5530

^1^ The pH of fibers was measured after ten times dilution by distilled water.

**Table 2:** Composition of culture and cementation solutions.

Solution	Substance	Amount (g/L)
Culture medium	Tris-buffer	1.575
	Ammonium sulfate	1
	Yeast extract	2
Cementation solution	CaCl2	5.55
	Urea	3
	Nutrient broth	0.3

## Data Availability

Data available on request.
